# The decline of adult smallpox in eighteenth-century London^1^

**DOI:** 10.1111/j.1468-0289.2011.00599.x

**Published:** 2011-11

**Authors:** Romola Davenport, Leonard Schwarz, Jeremy Boulton

## Abstract

Smallpox was probably the single most lethal disease in eighteenth-century Britain, but was a minor cause of death by the mid-nineteenth century. Although vaccination was crucial to the decline of smallpox, especially in urban areas, from the beginning of the nineteenth century, it remains disputed the extent to which smallpox mortality declined before vaccination. Analysis of age-specific changes in smallpox burials within the large west London parish of St Martin-in-the-Fields revealed a precipitous reduction in adult smallpox risk from the 1770s, and this pattern was duplicated in the east London parish of St Dunstan's. Most adult smallpox victims were rural migrants, and such a drop in their susceptibility is consistent with a sudden increase in exposure to smallpox in rural areas. We investigated whether this was due to the spread of inoculation, or an increase in smallpox transmission, using changes in the age patterns of child smallpox burials. Smallpox mortality rose among infants, and smallpox burials became concentrated at the youngest ages, suggesting a sudden increase in infectiousness of the smallpox virus. Such a change intensified the process of smallpox endemicization in the English population, but also made cities substantially safer for young adult migrants.

The late eighteenth century was a crucial period in English population history, marking the beginning of the demographic transition. On the mortality side, the period saw a rise in life expectancy that was moderate in rural areas but resulted in the transformation of the urban mortality regime. London in particular ceased to function as a brake on the national population, consuming the population growth of the countryside, and became a self-sustaining centre in which births exceeded deaths.^2^ This period also witnessed the emergence of class differences in mortality, heralding the modern mortality regime where urban and higher socioeconomic groups enjoy significantly higher survival chances than their rural and poorer peers.^3^ Despite the huge significance of this period for our understanding of population growth and mortality decline, we still know little more than the bare outline of events. While the work of the Cambridge Group has provided very detailed information on the age structure of mortality decline through the technique of family reconstitution, we know very little about the changes in disease patterns that were the proximate cause of these changes, because parish data rarely included information on cause of death.^4^ Moreover, the reconstituted populations did not include any large towns, and it is clear that early modern cities, and especially London, had very different mortality regimes from rural areas, and experienced more profound changes in the late eighteenth and early nineteenth centuries. However, although we know relatively little about changes in death rates in urban areas, almost all we know of causes of death in this period comes from urban populations, because the main sources for London and several other large towns, the bills of mortality, include information on cause of death. Used with caution, urban cause of death data offer a rare insight into epidemiological changes in the national as well as urban populations, both because cities served as disseminators of epidemic diseases, and because urban populations often contained large numbers of rural migrants. In the case of London, the Bills of Mortality indicate that smallpox was probably the single most lethal cause of death in the eighteenth century, accounting for 6–10 per cent of all burials. However, by the 1840s smallpox was a minor cause of death, suggesting that the decline of smallpox mortality played a major role in the reduction of all-cause mortality, at least in urban areas. In this article we discuss evidence from a novel source of mortality data, the sextons' books of the large London parish of St Martin-in-the-Fields, which allows us to follow age-specific changes in smallpox burials, and provides new insight into smallpox mortality in both London and its migrant hinterland.^5^ We use these data to test the hypotheses that a decline in smallpox mortality occurred in the late eighteenth century (before vaccination), and that changes in smallpox mortality rates were a major factor in the decline of mortality in both rural and urban areas during this period.

## I

Smallpox was highly lethal (killing one-seventh to one-quarter of its victims),^6^ and conferred lifelong immunity on survivors. As such it was subject to the process of endemicization, appearing initially as an infrequent epidemic disease affecting all ages, but returning more frequently as population densities and interactions increased. As the frequency of epidemics increased, a growing proportion of the adult population would have acquired immunity to the disease, and smallpox was clearly a childhood disease in the London-born population by the eighteenth century, always present, but peaking every two to three years.^7^ However, much of the London population consisted of migrants, some of whom had not encountered smallpox in childhood, and so, despite the endemic nature of smallpox in London, adults still comprised a significant share of smallpox victims, reflecting the incomplete endemicization of smallpox outside the metropolis.^8^

**Figure 1:**
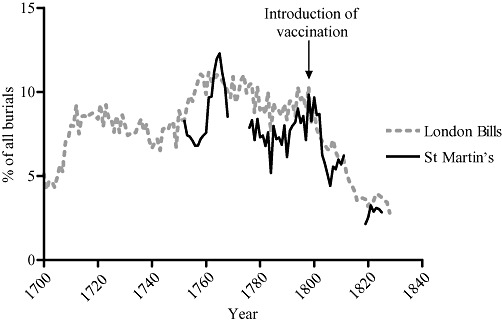
Smallpox as % of all burials (five-year moving means). *Sources:* Marshall, *Mortality*, unpaginated tabs.; CWAC, London, Accession 419/123, 233–244, F2469, St Martin-in-the-Fields, sextons' day books.

Smallpox mortality seems to have peaked in London in the 1760s, before declining slowly, and then rapidly after 1800 (both as a proportion of burials, and as absolute numbers of burials reported in the London Bills) (figure 1). The rapid decline in smallpox burials after 1800 coincided with the widespread adoption of Jenner's cowpox vaccination method, and despite incomplete coverage and low levels of re-vaccination, vaccination programmes succeeded in reducing smallpox to a relatively minor cause of death by the beginning of civil registration in 1837. However, the cause of the slower decline in smallpox burial totals before 1800 remains obscure. The practice of inoculation (deliberate infection with attenuated smallpox virus to confer immunity) became popular in the 1760s with the introduction of a safer and more effective procedure (known as the ‘Suttonian method’).^9^ Razzell has argued that inoculation was widely practised from the 1760s and had a spectacular effect in reducing mortality, although he found little evidence of inoculation being practised in London itself.^10^ It is also possible that smallpox underwent a natural decline in virulence, although the scanty case-fatality data that exist for England suggest the opposite.^11^

To interpret the behaviour of smallpox in London and its migrant hinterland, we need to know the age structure of smallpox burials. The London Bills provide burials by age and by cause separately, but not cross-tabulated, so we only know the smallpox burial totals for all ages. However, Landers was able to analyse the age structure of smallpox deaths within a subsection of the London population using Quaker burial registers.^12^ The registers included age and cause of death information, and indicated that adults (aged 10 and over) were at high risk of smallpox in London, accounting for nearly 40 per cent of smallpox burials in the second half of the seventeenth century, and 22 per cent in the period 1750–99. Landers was unable to trace any adult smallpox death to a London baptism, and concluded that adult smallpox victims were almost exclusively migrants to London. Smallpox rose dramatically as a proportion of all causes of death in the first half of the eighteenth century, but then declined somewhat in the second half of the century. Landers was able to demonstrate that much of the rise in childhood mortality (ages 1–9) in the Quaker sample in the early eighteenth century, and its rapid fall especially from the 1770s, was due to the rise and fall of smallpox mortality at these ages.^13^ Older infants (aged 6–11 months) also experienced a decline in smallpox mortality in the later eighteenth century, and this contributed to the dramatic reduction in infant mortality in this period. Infant mortality among the London Quakers declined from very high levels of around 350 per 1,000 births in the 1740s to below the national average of *c.* 160 per 1,000 in the 1840s, and although the decline was dominated by reductions in early infant deaths (attributed to mainly non-infectious ‘endogenous’ causes), there was also a significant reduction in infectious disease (or ‘exogenous’) infant mortality.^14^ This is in contrast to the national picture derived from the Cambridge Group reconstitution sample, which indicated that the notable improvement in infant mortality rates from the 1770s was due almost exclusively to improvements in endogenous infant mortality, with little improvement in the mortality rates of older infants.^15^ This raises the question of whether infants and children in urban areas enjoyed reductions in infectious disease mortality similar to the Quakers in this period, which would have promoted more rapid gains in life expectancy in urban areas.^16^

Landers attributed the decline in smallpox mortality before 1800 in his London Quaker sample primarily to the adoption of inoculation. However, although Landers suggested that a reduction in smallpox also played a role in the notable improvement of survival chances of children in the capital more generally, he hesitated to attribute this to inoculation, because of the paucity of evidence for widespread inoculation within London itself.^17^ Although infant mortality appears from the evidence of the London Bills to have fallen precipitously in the period 1770–1830, it is not possible from the Bills to determine anything about the age pattern of mortality declines within the first year of life. Therefore it remains unknown whether infant mortality in the wider population declined with a pattern similar to that of Quaker infants, and whether smallpox played such a significant role. The Quaker sample suffers from several limitations that make it an equivocal guide to changes in the wider London population. The small size of the Quaker sample (perhaps 1,000 individuals at its peak) precluded a fine temporal analysis of the cause-specific data, and the smallpox deaths were analysed by 50-year periods.^18^ In addition, it is probable that the Quakers differed from the rest of the population in composition and behaviours that made their mortality patterns unrepresentative of the wider metropolitan population in some respects. Infant mortality of Quakers showed some unusual characteristics, being notable for unusually low levels of endogenous mortality even at the start of the period,^19^ and it is possible that the London Quakers adopted smallpox inoculation with more enthusiasm than the rest of the metropolitan population.^20^

The late eighteenth-century decline of smallpox that is apparent in the London Bills and in Landers's reconstitution sample of London Quakers coincided with a surge in national population growth, and dramatic improvements in particularly urban death rates. Several authors have argued for a pre-eminent role for inoculation and later vaccination in reducing mortality in the eighteenth and early nineteenth centuries.^21^ However, the roles of smallpox and of medical measures to curb its destructiveness have been rather neglected in the heated debate regarding the drivers of secular mortality decline. In particular, McKeown attempted to explain the late eighteenth-century decline in mortality by extrapolation from his analysis of the Registrar-General's cause of death data from 1838 onwards, and was apparently unaware of the magnitude of the decline in smallpox mortality in the intervening period, and so dismissed both inoculation and vaccination as making an insignificant contribution to mortality decline.^22^ The subsequent debate has tended to focus on the interpretation of the nineteenth-century sources on which McKeown relied, to the neglect of the earlier period of mortality decline, which had quite different characteristics.

In this article we present new evidence regarding smallpox in London, using age- and cause-specific data from the large parish of St Martin-in-the-Fields. This evidence is used to assess the extent of smallpox exposure in London's migration sphere, and to examine the causes of the apparent fall in smallpox mortality in the late eighteenth century, before vaccination. This study is pioneering in the sense that, unlike those who rely on the Bills of Mortality, we can look at the age-specific incidence of diseases in a large area of London from 1752 to 1805. Our evidence derives from the sextons' records of burials, giving the cause of death, age, name, address, and burial fee of almost everyone who was buried in the parish.^23^ These thus make it possible to quantify the incidence and location of smallpox at the heart of England and in Europe's largest city. St Martin's was a large Westminster parish, with probably some 25–30,000 inhabitants throughout the course of the eighteenth century.^24^ Westminster had, even by London standards, a high proportion of recent migrants.^25^ These migrants were predominantly young adults, with a high proportion of women in domestic service. The sextons' reports for the parish are remarkably complete. There is some omission of age and cause of death, but such omissions appear to be random. Exact age at death is given, and although there is considerable age heaping at older adult ages, this does not affect the ages at which most smallpox burials occurred. Importantly, age at death was recorded in days, weeks, and months for infants, allowing fine-grained analysis of the age structure of mortality in infants and young children. Although most causes of death are problematic to identify in this period, smallpox was easy to recognize, and was probably reported fairly accurately.^26^ The only systemic bias was with fulminating smallpox, which killed before the pock-marks appeared, and was most common in infants.^27^ Smallpox could be confused with severe chickenpox, but the latter was very rarely lethal. Where possible, totals of smallpox burials and burials by age have been corrected for missing ages and causes, but such corrections had little effect on the conclusions, given the lack of bias in these omissions. Although the dataset covers the period to 1825, data for years after 1805 have been omitted, because the workhouse burials were moved elsewhere from 1806, and although these records have been retrieved, they lack information on cause of death.

Section II describes the patterns of smallpox mortality in St Martin's, in particular the rapid decline in adult smallpox burials after *c*. 1770. Section III considers the composition of adult smallpox victims, especially with respect to their geographical origins. Section IV uses the changes in age patterns of smallpox in children to evaluate the contributions of inoculation and endemicization to the decline in adult smallpox in London, and the implications of these findings are summarized in section V.

## II

Smallpox burial totals in St Martin's resembled very closely the pattern evident in the London Bills as a whole, with a maximum in the 1760s and a very rapid decline after 1800 (figure 1). Table 1 gives the ages of smallpox deaths in St Martin's, in the period before vaccination. It must be stressed that this information is virtually unique for this period, being based on large sample sizes with little bias in omission by age and cause.

**Table 1:** Percentage age distribution of burials from smallpox in St Martin-in-the-Fields

Age	1752–66	1775–99
0	13.7	23.3
1–4	54.5	61.5
5–9	10.9	9.4
10–19	4.6	1.8
20–49	15.6	3.5
50+	0.7	0.6
Mean age at death (years)	7.8	3.9
*N*	1,083	2,022

*Notes:* The periods include only years where burials without exact ages formed less than 5% of the total. Both age and cause of death were poorly recorded in the period 1767–74. Years after 1799 were excluded, to exclude effects of vaccination. Cause of death was given for 99% of burials in the period 1752–66, and 92% of burials in the years 1775–99, partly as a consequence of the inclusion of exported burials (see below, n. 66). Smallpox burials were adjusted for burials of unknown age and cause. Most burials without exact age were designated as child (‘C’) or adult (‘M’ or ‘F’) in the sextons' books, and almost all child burials were aged under 10, where exact age was given. Where cause was given but not exact age, burials were distributed to exact ages using the cause-specific distribution of burials by age for age groups under 10 or 10 and over. Burials with no cause of death given were first distributed to exact ages, and then distributed according to the age-specific ratio of smallpox burials to other causes. Almost all smallpox burials included exact age, and there was little age bias among burials with no given cause. Therefore the patterns produced by the redistribution of burials of unknown age and cause did not differ significantly from those of unadjusted smallpox burials. This is in contrast to Landers's analysis of London Quakers, where the redistribution of deaths of unknown cause caused large changes in the age patterns of smallpox burials especially at younger ages; Landers, *Death*, pp. 153–4. Both Landers's and the current analysis assumed that the risk of omission of cause of death was independent of the cause. This assumption is not critical in the case of St Martin's, because the adjusted series is not very different from the unadjusted (using only burials explicitly described as smallpox victims). However, if smallpox were more likely to be recorded than other causes, this may invalidate some of Landers's conclusions.

*Source:* CWAC, London, Accession 419/123, 233–244, F2469, St Martin-in-the-Fields, sextons' day books.

The contrast between the two periods, 1752–66 and 1775–99, is obvious. The effect of the influx of young people to London is clear *only* in the earlier period. There is a large bulge of deaths at ages 20–49, which is inconsistent with the exponential decline in mortality predicted if smallpox were endemic. What is remarkable is that this is almost absent in the last quarter of the century. This represents a key finding of this study. The decline in smallpox was associated with a dramatic decline in smallpox deaths among adults, and a concentration among children aged under five.

**Figure 2:**
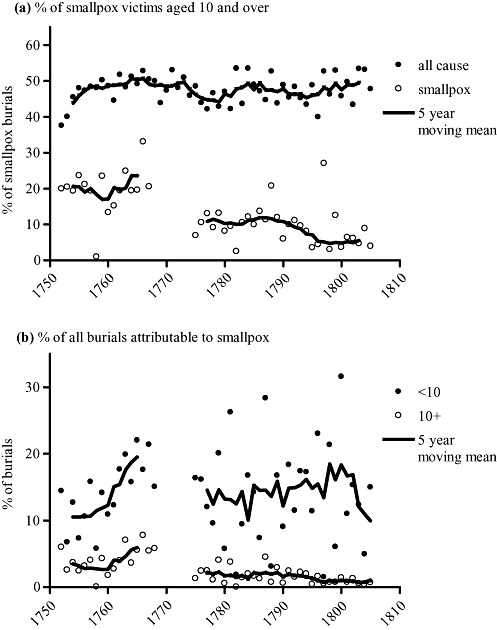
St Martin-in-the-Fields: (a) % of smallpox victims aged 10 and over; (b) % of all burials attributable to smallpox. *Notes:* Smallpox and all-cause burials were adjusted for burials with missing ages and missing causes, as described in tab. 1. The period of poor recording of age and cause, 1767–74 (see note to tab. 1), is evident in the smallpox plots (whereas ages could be redistributed for all-cause mortality without serious distortions), and moving five-year means were fitted only where a complete five-year window existed. *Source:* As for tab. 1.

The large size of the St Martin's population makes it possible to analyse the smallpox burials by single years, and thus to pinpoint the period when this dramatic age shift occurred. Figure 2a shows the proportion of smallpox burials attributed to adults (corrected for missing causes and ages). A feature of the decline is its rapidity. Adults halved as a proportion of smallpox burials (from 20 per cent of all smallpox deaths to 10 per cent) in an eight-year period, 1767–74. Although this decline coincided with a period of poor recording of age and cause in the sextons' books,^28^ there was no corresponding change in the proportion of adults dying from all causes, so the decline in adult smallpox deaths cannot be attributed to some change in age recording or a precipitous decline in adult immigration.^29^ Rather, figure 2b indicates that smallpox ceased to constitute a major risk to adults. Smallpox accounted for 2–4 per cent of adult burials in the 1750s, but averaged only 1 per cent after the mid-1770s. The reduction in adult smallpox burials was preceded by a rise in smallpox mortality in the 1760s, as indicated by an absolute rise in smallpox burials in London, as figure 1 shows, and a rise in the proportion of burials attributed to smallpox among both children and adults in St Martin's (figure 2b). Also notable is that the decline in adult smallpox mortality coincided with an increase in the importance of smallpox as a cause of death in children (accounting for 14–15 per cent of burials in the last quarter of the eighteenth century, compared with *c*. 11 per cent in the 1750s: figure 2b).

Since this phenomenon of precipitous changes in the age structure of smallpox mortality appears in a local study, we need to establish that it is not a product of some local peculiarity in the care of smallpox sufferers. Were adult burials being sent outside the parish to institutions? One institution designed for just this purpose was the London Smallpox Hospital, established in 1746. It admitted only persons over the age of seven and recommended by one of the subscribers.^30^ Between 1746 and 1763 the hospital admitted 6,456 persons, or an average of 359 a year; from 1776 to 1800 it took 7,017 persons, or an average of 280 a year, a miniscule number in a city the size of London.^31^ Since the case fatality rate in the hospital was only about a quarter, the Smallpox Hospital cannot have removed more than a hundred or so victims per year from the entire metropolis, and thus cannot explain the disappearance of adult smallpox victims.

**Figure 3:**
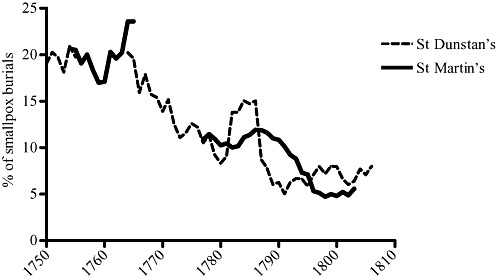
Proportion of smallpox deaths aged 10 and over, five-year moving average, St Dunstan's, Stepney, and St Martin-in-the-Fields. *Notes:* St Martin's smallpox burials are adjusted for burials with missing ages and missing causes, as described in tab. 1. In the case of St Dunstan's only smallpox burials were transcribed from the sextons' books, so these have been corrected for missing ages but not unknown causes. Cause of death data were missing for the years 1758–61 in the St Dunstan's sextons' books. Decadal smallpox burial totals for St Dunstan's were 391 (1740–9), 211 (1750–7), 385 (1762–9), 526 (1770–9), 346 (1780–9), and 327 (1790–9) (total 2,186 burials). *Sources:* London Metropolitan Archives, P93/DUN/173-192, St Dunstan's, Stepney, sextons' day books; as for tab. 1.

However, to be sure that St Martin's was indeed typical, it was necessary to study another parish. Fortunately, it was possible to do this for the large east London parish of St Dunstan, Stepney. The parish had a larger population than St Martin's—about 40,000 in 1801—and covered a very different, and poorer, part of the capital.^32^ A reasonably complete set of its sextons' records has survived. Figure 3 shows that the same trend was at work. Different in so many ways, the two parishes are remarkably similar as far as smallpox was concerned.

We can therefore conclude that smallpox remained a significant threat to adults in London before the 1770s, but was confined largely to childhood thereafter. Such a change in the nature of smallpox mortality in London is corroborated by various aspects of Landers's analysis of the London Bills. He found smallpox burials to be associated with child and young adult burials before 1775, but more closely with child burials subsequently.^33^ Moreover, before the 1770s smallpox mortality was positively associated with conditions that favoured migration of especially young adult males into the capital, specifically demobilization of armed forces after conflicts.^34^ From the 1770s onwards these associations ceased to be significant (although they remained significantly associated with fever mortality, indicating that the migratory patterns supposedly underlying these phenomena probably had not changed).^35^

The precipitous decline we have documented in adult smallpox in the 1770s, in the absence of any other evidence of a sudden shift in the age structure or migration patterns in the St Martin's population, strongly suggests that adult migrants were increasingly immune to smallpox. Such immunity among London's migrants could have been due to either: (1) an increase in childhood exposure to smallpox throughout London's migrant catchment area; or (2) inoculation and later vaccination of virtually all London migrants; or both.

We will evaluate the contribution of each of these processes in section IV, following an examination of the characteristics of adult smallpox victims, and the state of our knowledge regarding the endemicization of smallpox in eighteenth-century Britain, in section III.

## III

The existence of large numbers of adult smallpox victims in London before the 1770s is evidence of the infrequent appearance of smallpox in parts of the English population in this period. Outside cities, cause of death data are very limited, and therefore our knowledge of the geography of smallpox epidemics and mortality remains extremely fragmentary. Creighton considered smallpox to be ‘exclusively an affair of childhood’ in large provincial towns, and attributed the high proportion of adult smallpox victims in London to its peculiarity ‘in receiving a constant recruit direct from the country’, where he supposed smallpox to be infrequent.^36^ Razzell provides some evidence of a north–south divide in smallpox behaviour, with smallpox seemingly a childhood disease even in small northern settlements by the mid-eighteenth century, but still an infrequent epidemic disease affecting all ages in some southern market towns.^37^ For example, in Kilmarnock, Scotland, 94 per cent of smallpox victims between 1728 and 1763 were aged under seven, and in Manchester only one adult (aged over 10) was recorded as dying, of 589 smallpox victims in the period 1769–74.^38^ If correct, these figures imply that even most migrants to these northern towns were immune to smallpox. By contrast, a smallpox outbreak in the southern market town of Burford in 1758 caused very high mortality among adults as well as children, with perhaps less than 40 per cent of deaths occurring among children under 10.^39^ A smallpox ‘census’ of Stratford-upon-Avon in 1765, taken to ascertain the numbers of inhabitants vulnerable to smallpox, indicated that many adults lacked immunity.^40^ In Cuxham, Oxfordshire, adults comprised nearly 30 per cent of those vulnerable to smallpox during an epidemic in 1772.^41^ Dobson concluded in an impressionistic survey of smallpox in south-eastern England that smallpox epidemics were a periodic feature of market towns, whereas isolated upland settlements experienced more irregular outbreaks, often affecting all ages.^42^ From these and similar fragmentary sources of evidence^43^ it seems likely that at least in the mainly southern communities from which most migrants to London were drawn,^44^ smallpox was not always a childhood disease by the mid-eighteenth century. This contrasts with contemporary claims that smallpox was endemic in the dispersed populations of mainland Scotland, and vital registration evidence indicating that smallpox was a childhood disease in most of rural Sweden at least from the mid-eighteenth century.^45^ While the reasons for the persistence of adult susceptibility in the south of England are unclear,^46^ it seems plausible that migrants from these southern settlements accounted for the bulge of adolescent and young adult smallpox burials in London. Where smallpox was endemic, adults (those aged 10 and over) comprised less than 10 per cent of all smallpox burials.^47^ Although London had an unusually high proportion of adults in the population, the constant influx of young adult immigrants is not sufficient in itself to account for the bulge of smallpox burials at these ages, without the further assumption that young adult immigrants were at higher risk of smallpox.^48^

So far we have assumed that adult smallpox victims were predominantly migrants. Smallpox was clearly endemic in London, and children under five were the main victims. Few native Londoners would have survived to adulthood without encountering smallpox. Writing in 1781, William Black observed:

I am induced by various considerations to believe that whatever share of smallpox mortality takes place in London amongst persons turned of twenty years of age, is almost solely confined to the new annual settlers or recruits, who are necessary to repair the waste of London, and the majority of whom arrive in the capital from twenty to forty years of age.^49^

Theory suggests that adult smallpox victims were mainly recent migrants from areas where smallpox was still an infrequent epidemic disease. Landers identified demobilized young males, and more generally subsistence migrants, as the main candidates. Both Landers and Galloway, using different statistical techniques, found strong associations between smallpox mortality and wheat prices, and poor weather, suggesting a connection between migration into London during periods of rural dearth and elevated smallpox mortality.^50^ Duncan et al. have argued with respect to smallpox as well as other diseases that fluctuations in wheat prices triggered regular epidemics, although these effects were not significant in late eighteenth-century London.^51^

The information on sex and burial fees in the sextons' books allows us to test these propositions with respect to St Martin's. The St Martin's population was heavily female-biased, due to the predominance of women in domestic service, and this is reflected in the low sex ratio (male to female) of burials at all adult ages. However, smallpox burials showed a slight male excess at young adult ages before the 1770s, in stark contrast to the female excess recorded for other causes of death at these ages in table 2. After 1774 the pattern changed markedly, to one resembling the low sex ratio of other causes of death. At the same time the adult proportion of smallpox burials dropped after the 1760s to levels typical in populations where smallpox was endemic (*c.* 5 per cent).^52^ Such a change in the sex ratio of smallpox burials is consistent with Landers's hypothesis that military recruits from areas where smallpox was infrequent could have swelled the numbers of adult smallpox victims before the 1770s. However, there was no evidence that adult smallpox victims were more likely to be poor (that is, subsistence migrants). In fact both child and adult smallpox burials were less likely to be pauper than were burials from other causes, and table 2 shows that this relationship was especially strong before 1775. Conversely, few adult smallpox burials commanded the highest burial fees, and therefore the average cost of non-pauper burials was slightly lower for smallpox than for other causes. Interestingly, Meier reported a similar distribution of adult smallpox burials by cost for St Martin's in the late seventeenth century, and a predominance of males among adult burials.^53^ Thus the migrants who comprised the majority of adult smallpox victims before 1775 were typically neither destitute, nor predominantly female domestic servants.

**Table 2:** Sex ratios and cost of burial for adults aged 10–39, by cause and period

Period	Other causes	Smallpox
*Sex ratio (males/100 females)*		
1752–66	65.40	121.35**
1775–99	79.26	75.56
*% paupers*		
1752–66	45.87	26.40**
1775–99	41.10	31.65
*Geometric mean cost of non-pauper burials (pence)*		
1752–66	355.79	325.33
1775–99	306.08	268.93
*Sample size*		
1752–66	1912	197
1775–99	3180	79

*Notes:* Exported burials were excluded from analysis, because these all incurred a uniform fee. ‘Other causes’ included burials where no cause was given (their inclusion had little effect on the results). Geometric mean cost was calculated for non-pauper burials to normalize the distribution of costs. Asterisks indicate statistical significance of differences between smallpox and other causes: * *p* < 0.05, **p < 0.01. Statistical tests used were Fisher's exact test for differences in proportions (sex ratios and proportions pauper), and one-way ANOVA tests for differences in geometric mean cost. Sample sizes refer to the samples used for calculations of sex ratios and % of pauper.

*Source:* As for tab. 1.

These findings are intriguing. The evidence that females were at lower risk than males before the 1770s is consistent with the suggestion that female servants in London were more likely than males to have originated from or to have migrated via other urban centres, where they would probably have encountered smallpox if they had not before.^54^ Moreover, there is evidence of a preference of some employers for domestic servants with evidence of smallpox scarring or inoculation.^55^ If such a preference exerted any selective effect on migrants for domestic service, then female migrants, for whom domestic service was the most common occupation, may have been more likely than male migrants to be immune to smallpox infection. Unfortunately the sextons' books do not contain occupational data, so it is not possible to determine whether smallpox victims were more likely to belong to certain occupational groups, such as apprentices or artisans, who might have been drawn from a wider geographical sphere than domestic servants, which included more isolated areas.^56^ St Martin's was also a point of entry for elite families, residing in London for the Season and drawn from all over the country. However, very few smallpox burials were in the highest quintile of burial fees, so this particular group of migrants cannot account for the curious patterns of smallpox burials by sex and status before the 1770s.

After 1775, the changes in the proportion and sex ratio of adult smallpox burials suggest that there was no longer any distinction in smallpox risk between the London-born and immigrant populations. These dramatic changes in the pattern of adult smallpox deaths might point to widespread inoculation, or to an increase in the circulation of the disease outside London. Section IV evaluates these possibilities.

## IV

In this section we first describe the possible roles of inoculation and endemicization in reducing the number of adult smallpox victims in London. We then test these two alternatives using the age patterns of child smallpox burials. Razzell is the foremost exponent of the importance of inoculation in the eighteenth century, and dates its rise in popularity, especially outside London, to the 1760s.^57^ He documents clear declines in smallpox as a proportion of burials in a number of parishes following mass inoculations, and some of these events are dated to the 1760s and 1770s. Writing in 1778 the physician Dimsdale anticipated a decline in smallpox mortality in London as a consequence of the spread of inoculation in the counties surrounding London and among ‘such inferior persons as may be supposed to supply London’.^58^ The coincidence in timing is striking, although the evidence for the scale and rapidity of the uptake of inoculation is too patchy to be confident that it occurred on a scale sufficient to explain the sudden drop in adult smallpox burials in London, within a seven-year period. Nonetheless, it is possible that inoculation was adopted with particular avidity by would-be migrants, as suggested by this treatise of 1767:

No sooner are the lower sort recovered [from inoculation], but they aim (the women especially) to get a servitude in London, or to use their own words *to better themselves*; this is the only objection that can be made to inoculation, and indeed it is one, for before they did not dare to quit the place of their birth for fear of that distemper, so remained honest and useful in the country.^59^

The anonymous author of this treatise was so splenetic towards the lower orders that his objectivity must be in considerable doubt; however, the passage might suggest that, like modern-day travellers from the developed world to Third World countries today, inoculation became part of the preparation of the sensible London migrant.

Once *in* London, the evidence is inconclusive. Dealing with specialized institutions that provided inoculations to adults is straightforward. Within London there was only the London Smallpox Hospital, founded in 1746, but this only provided some 632 inoculations in an average year between 1746 and 1832.^60^ The physician and philanthropist John Coakley Lettsom sought to establish a society for inoculating the London poor in their homes in 1775, presumably adults as well as children. It failed, partly owing to opposition from the eminent physician Baron Thomas Dimsdale, who was himself a prolific inoculator, but who feared the consequences of introducing infection to London—as if smallpox were not already endemic there. Popular indifference to inoculation was probably also a factor in the failure of the scheme. Lettsom tried again and failed in 1779.^61^

There was, however, one set of institutions in London that did inoculate poor children, whatever their parents thought, and these were some parish workhouses and the Foundling Hospital.^62^ The Foundling Hospital was doing this already in 1743, and in 1749 the governors advertised that all the children who had not had smallpox when at nurse would be inoculated on their return to London.^63^ The parish of St James, Westminster, entered it in their standing orders in 1756:

All the Children are inoculated for the Small-Pox when deemed proper by the Surgeon, and he is paid Ten Shillings and Sixpence for every Child that survives that Disorder. The Nurse is likewise paid Ten Shillings and Sixpence for every child that has it in the natural Way, or is inoculated and survives, but not else.^64^

It is likely that a number of other London parishes did this, and the documentation may come to light. However, the London workhouses, large though they were, served only a small proportion of a parish's poor, and many of these never reached adulthood.

Although inoculation remained unpopular in London in the eighteenth century, it remains possible that the rural surge in popularity of inoculation in the 1760s induced many young adults to seek inoculation before migration to London, and this was sufficient to produce a dramatic drop in the numbers of adult smallpox victims in this period.

An alternative explanation is a rise in the level of smallpox exposure in London's migration sphere, resulting in the infection of most individuals before migration. Such a rise in the frequency of smallpox epidemics, which would lower the average age at infection, could occur as a consequence of an increase in population density or connectedness. This would result in more frequent contacts between infected and non-immune or ‘susceptible’ individuals, and an increase in the frequency of smallpox transmission between and within communities. Alternatively the virus could have changed its biological properties, becoming more infectious and therefore able to spread more easily for a given level of contact and population size.

No rise in population density in rural areas could account for such an abrupt decrease in the number of susceptible adults in London. However, transport links were growing at extraordinary rates in this period, and it remains possible that many areas of high emigration and low epidemic frequency became integrated very rapidly into a national network of disease transmission with which they had previously maintained only tenuous links.

So far we have described the processes by which inoculation or increased smallpox exposure could have acted to reduce the adult risk of smallpox in London, but we cannot distinguish between the two possibilities. However, while both processes would have had similar effects on adult smallpox rates, they should have produced contrary effects if any on childhood smallpox mortality rates. The spread of inoculation should have had no effect (if inoculation was confined mainly to adult migrants) or reduced childhood rates (if children within London were inoculated). An increase in smallpox transmission outside London would be expected to produce the opposite result: childhood smallpox rates should have been unaffected, or should have increased (if the London population became denser too, or if the smallpox virus itself became more infectious).^65^ In addition, rural inoculation should not have affected the age pattern of smallpox mortality among children in London, whereas an increase in transmission of smallpox should have reduced the average age at infection in rural areas, and could have had a similar effect in London, if the same processes were at work in the metropolis. Therefore it is possible to use the smallpox burials of children to test competing hypotheses regarding the virtual disappearance of adult smallpox. We have assumed that most children under 10 were London-born, but this is not a critical assumption.

**Figure 4:**
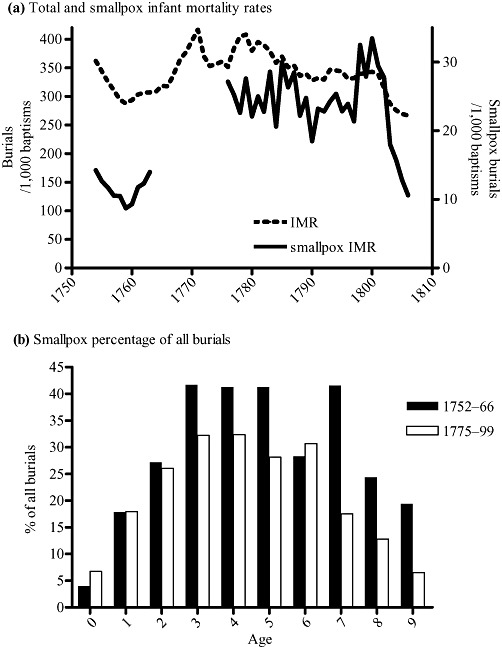
Smallpox in infants and children, St Martin-in-the-Fields. *Note:* Smallpox and all-cause burials were adjusted for missing age (and cause in the case of smallpox). IMR: infant mortality rate, or infant deaths per 1,000 births, calculated here as infant burials per 1,000 baptisms. *Source:* As for tab. 1.

**Figure 5:**
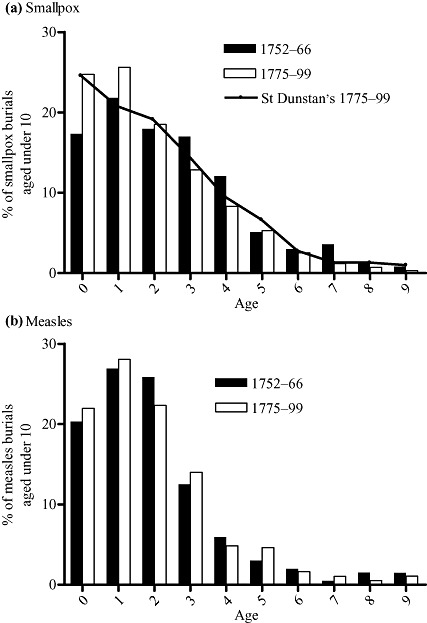
Age distribution of measles and smallpox deaths at ages under 10 (as % of burials from each cause aged under 10). *Note:* Measles and smallpox burials for St Martin's were adjusted for unknown ages and causes (see note to tab. 1). The St Dunstan's burials were adjusted for unknown ages. *Sources:* As for fig. 3.

The evidence from St Martin's indicates that smallpox rose in importance among children in parallel with its decline in adults. Smallpox accounted for around 11 per cent of burials aged under 10 in the 1750s, before peaking, in concert with adult burials, at *c.* 20 per cent in the 1760s (figure 2b). Smallpox then declined to roughly half its 1750s level as a cause of death in adults, but remained above the 1750s level, at around 14 per cent, in children. Moreover, in the period when adult smallpox burials halved, the age distribution of smallpox burials among children under 10 also shifted to younger ages. In particular, the infant smallpox mortality rate doubled, while rates in older children probably declined. Figure 4a shows the total and smallpox infant mortality rates per 1,000 baptisms. While there was some increase in the total infant mortality rate,^66^ the smallpox rate doubled, from around 15 to 30 deaths per 1,000 baptisms from the mid-1770s. Smallpox also rose from 4 per cent of burials to account for almost 7 per cent of burials in the first year of life (figure 4b). At the same time, smallpox declined as a proportion of burials in older children (aged three and over), suggesting increased levels of immunity at these ages. As a consequence, the distribution of smallpox burials at ages under 10 became concentrated at the youngest ages after 1770, as figure 5a shows.

These changes in smallpox burial patterns among children—a rise in the importance of smallpox as a cause of death in infancy, and a reduction in the average age of death—are consistent with an increase in smallpox transmission rates, and are difficult to explain as a consequence of inoculation. In particular, it is difficult to see how protection of adults and/or children by inoculation could have increased the infant smallpox death rate. However, care must be exercised in interpreting these data. The total infant mortality rate in figure 4a is very high, suggesting an under-recording of baptisms particularly in the last quarter of the century. Moreover, burial totals fluctuated as a consequence of the London market in burial spaces, and so the infant mortality rates calculated here are unreliable as a guide to the real trends in death rates.^67^ Nevertheless, there was no evidence of any discontinuities in the age patterns of burials, and in particular there was no evidence of any bias in the recording of smallpox burials that would have caused such a great increase specifically in smallpox burials compared to other causes.^68^ However, the ratio of burials in early infancy to burials in later infancy declined over the period, and it is possible that this reflected in part a deterioration in the recording of neonatal deaths. Since smallpox mortality was higher in the later months of the first year of life, deficiencies in recording of deaths in early infancy, or improvements in mortality specifically at those ages, could have exaggerated the importance of smallpox as a cause of infant death (although these could not account for the doubling of the smallpox infant mortality *rate*). Table 3 presents smallpox burials as a proportion of all burials in early and late infancy (the first and second six months of life). In both age groups smallpox burials increased by nearly 50 per cent as a proportion of all burials, indicating that the rise in importance of smallpox as a cause of death in infancy was not simply a consequence of a shift in the pattern of burials between early and late infancy. Notably, even very young infants were at risk of smallpox, because although maternal smallpox antibodies transferred *in utero* conferred immunity to infants of immune mothers, this immunity waned quickly, regardless of breastfeeding.^69^ Thus at high levels of infectiousness and epidemic frequency, smallpox mortality could become concentrated in infancy (as indicated in table 1, where infants accounted for almost a quarter of smallpox burials in the period 1775–99). Indeed, even these very high estimates of the proportions of infant smallpox victims probably underestimate the impact of smallpox in infancy. Lettsom considered the true smallpox rate in London to be double that recorded in the Bills, due to the omission of large numbers of infant smallpox deaths, that were assigned incorrectly to ‘convulsions’.^70^ Infants were probably particularly susceptible to fulminating smallpox, and such cases generally would have been attributed to some other cause, particularly the ubiquitous ‘convulsions’.^71^ Therefore it is probable that any shift in the average age of infection to younger ages would have resulted in a greater level of under-recording of smallpox mortality.

**Table 3:** Percentage of all burials attributed to smallpox, by months of life, St Martin-in-the-Fields (total burials in age group in parentheses)

Period	0–5 months	6–11 months
1752–66	2.06 (2,979)	11.90 (724)
1775–99	2.99 (4,960)	16.68 (1,983)
% change	145	140

*Notes:* Smallpox burials were adjusted for missing cause, but not for missing age burials. The relatively small numbers of burials precluded further breakdown by age.

*Source:* As for tab. 1.

The rise in importance of smallpox in infants and decline at older ages is consistent with a reduction in the average age of infection. Such a reduction, in the London-born population, indicates an intensification of the endemicization process.^72^ While smallpox could have become more readily transmitted outside London as a consequence of rapid changes in transport networks, for instance, it is more difficult to account for the sudden changes in smallpox patterns in London-born children by such mechanisms. There is no evidence for any sudden increase in population density or family size within St Martin's, indicated, for example, by a rise in baptisms or an abrupt change in the age structure. Moreover, such changes would have had to occur simultaneously in London's east and west, to explain the synchronicity of changes in age patterns in St Martin's and Stepney. However, to test further whether any changes in either density or age structure of the population could have occurred, that would have caused a real or apparent reduction in the average burial age of child smallpox victims, we compared burials attributed to smallpox with those of measles.

Measles and smallpox are both viral infections spread by droplets and conferring long-lasting immunity on survivors, and are very similar with respect to ‘generation time’ (the average period between contact with an infectious person and onset of symptoms in the newly infected person).^73^ However, measles is more infectious, and therefore appears to have been more fully endemicized by the mid-eighteenth century. In St Martin's 99 per cent of all measles burials were aged under 10 by the mid-eighteenth century, and most deaths occurred at ages under five. By contrast, only 79 per cent of smallpox burials were aged under 10 in this period (1752–66), and child smallpox burials were less concentrated at the earliest ages. To examine changes in age at death among the London-born population, we again restricted the analysis to children under 10 years old, most of whom would have been born locally (figure 5a and b). In the last quarter of the eighteenth century measles showed little change in age pattern, indicating that there were no large changes in the age structure of the child population, and no significant rise in population density that facilitated viral transmission (the age distributions between the two periods did not differ significantly in a two-sample Kolmogorov-Smirnov test). However, the smallpox distribution shifted to resemble that of measles, with burials concentrated in the first two years of life. Indeed, smallpox burials became even more concentrated in the first year of life than was the case for measles, possibly because maternal-derived antibodies to measles persist for longer in the infant than antibodies to a number of other viruses, probably including smallpox.^74^ A similar pattern held in Stepney after 1773 (figure 5a),^75^ and in Sweden, where infants accounted for around 30 per cent of smallpox deaths in the last quarter of the eighteenth century.^76^ The shift in the age distribution of smallpox burials in childhood was substantial (*p* = <0.001 by two-sample Kolmogorov-Smirnov test), and, when contrasted with the lack of change in the age pattern of measles burials, indicates a genuine reduction in the age of death (and therefore age at infection) after 1775 that was specific to smallpox.

In the absence of any abrupt increase in population density, the most plausible explanation for the sudden rise in smallpox mortality at the youngest ages is an increase in infectiousness of the smallpox virus. Such a change could occur suddenly, through mutation or importation of a novel strain, and the more infectious strain would spread rapidly and displace less efficient strains. Obviously smallpox was already endemic in London by the mid-eighteenth century, as indicated by the concentration of smallpox deaths in early childhood, the weekly toll of smallpox burials, and the biannual cycle of epidemics. The high population densities and ease of mixing would have resulted in relatively efficient transmission of the virus, and the main effect of any increase in infectiousness would have been a further concentration of infection in infancy. The effect we have detected here is therefore rather small, given the already low age of childhood infection in London. However, the impact on small or isolated populations would have been much more profound. Smallpox transmission was relatively weak compared, for instance, to measles, and smallpox victims were usually only infectious once the symptoms were apparent, and were often too sick to move about.^77^ This probably made it possible for communities in southern England to avoid smallpox for years at a time (by luck and quarantining) and ultimately made it possible to eradicate smallpox globally with relatively low levels of vaccine coverage.^78^ An increase in infectiousness would have raised the chances of infection in infancy and early childhood in large urban populations, and at the same time promoted the circulation of smallpox in rural communities (by increasing the frequency of successful introduction of the virus, and reducing the effectiveness of quarantine). A higher rate of introduction, and more efficient transmission within a community (resulting in higher rates of export to other populations) may have resulted in the integration of small southern settlements into a national smallpox network. The period of sustained smallpox mortality in the 1760s may thus represent a period of adjustment, in which smallpox circulated more rapidly within the London population and enjoyed a large pool of child and young adult immigrant susceptibles. As smallpox became frequent in areas previously subject to only sporadic visitations, then the vulnerability of adult migrants would have declined, and smallpox would have become a disease of childhood in both London and its hinterland.

Other evidence for such a scenario, of increasing infectiousness, comes from case fatality rates. In smallpox, infectiousness may be associated with virulence (lethality), since both may depend on the number of viral particles produced.^79^ Razzell has argued for an increase in virulence over the eighteenth century, but the timing of such changes is very unclear.^80^ The case fatality rate at the London Smallpox Hospital rose from 255 per 1,000 between 1746 and 1763, to 320 per 1,000 between 1776 and 1800.^81^ In Boston, US, where inoculation was practised from the 1720s, fatality rates for the uninoculated rose abruptly after 1776.^82^ This apparent increase in lethality in the last quarter of the eighteenth century would be expected to be accompanied by an increase in transmission of smallpox.

The evidence for a shift in the infectiousness of the smallpox virus is tenuous, and depends on relatively small changes in the age pattern of mortality, raising the possibility that these changes are artifactual. The most serious problem in using burial records as evidence of mortality patterns is the lack of information on the ‘population at risk’, that is, how many people in each age group comprised the population from which the burials were derived. Changes in the absolute numbers of people at risk, or the relative sizes of different age groups, can cause significant changes in the numbers and age distribution of burials, without any real change in the age-specific rates (burials per 1,000 persons of a given age). In the case of St Martin's there were several factors that probably operated to cause fluctuations in the numbers at risk, including relatively high levels of migration, and a traffic in corpses for burial between parishes. Therefore we used several methods to test whether the observed patterns of smallpox burials reflected ‘real’ changes in smallpox mortality rates and age patterns. In addition to calculating smallpox burials as a proportion of all burials (to control for changes in burial totals), and comparing raw smallpox totals with those adjusted for missing causes and ages (which indicated little bias in the omission of cause and age), we also compared St Martin's with the parish of Stepney, where possible.^83^ As another type of internal check, we compared burials attributed to smallpox with those attributed to measles, which indicated that changes in the age pattern of smallpox burials could not be attributed to changes in the underlying age structure of the population at risk. Therefore we are confident that the changes in smallpox mortality that we have detected in St Martin's are genuine. However, our explanation for these phenomena, that smallpox became more infectious at some point around 1770, produces a number of other predictions regarding changes in smallpox mortality in populations outside London, which provide further tests of the plausibility of our hypothesis. Space does not permit a discussion of all of the available data, but the most interesting are from Geneva, where causes of death were recorded in an urban setting from 1580. Smallpox seems to have undergone several cycles of lethality in Geneva that are remarkably consistent with the fluctuations in smallpox burials in London.^84^ Although children were always the principal victims, Perrenoud notes a radical change in the age structure of smallpox burials from 1777. Infant smallpox rates tripled, and for the first time even neonates were affected, while rates in older children declined, causing Perrenoud to propose that a novel variant of the smallpox virus had arisen.^85^ The changes in the age pattern of smallpox mortality in Geneva are strikingly similar to those evident in the St Martin's population earlier in the 1770s, and consistent with the diffusion of a novel viral strain.

## V

Our finding that adults comprised a high proportion of London smallpox burials before the 1770s is consistent with previous findings, and indicates that smallpox remained an infrequent visitor within parts of London's migrant catchment areas by the mid-eighteenth century. Since adult smallpox burials did not appear to be typical of the female domestic servants that comprised the bulk of migrants in St Martin's, it remains an open question as to where these vulnerable adults originated. The pattern of endemicization of smallpox in England appears mysterious. Many northern settlements seem to have been almost free of adult smallpox by the mid-eighteenth century, yet a southern market town such as Burford could experience severe adult smallpox mortality in this period. However, we also discovered a very abrupt shift in the age pattern of smallpox burials to younger ages in the 1770s, in both St Martin's, Westminster, and St Dunstan's, Stepney. The existence of this phenomenon in two large and non-contiguous parishes, and the stability of other causes and age distributions of burials, indicate that this result is not an artefact. Rather it points to a sudden change in the pattern of infection in London's migration sphere. This change coincided with the spread of inoculation documented by Razzell, and inoculation doubtless contributed to the increased immunity of London migrants. However, inoculation alone could not account for the simultaneous shift in the age pattern of smallpox in children, most of whom would have been London-born. Smallpox became concentrated at the youngest ages, and appears to have become more destructive, the infant smallpox death rate almost doubling between 1766 and 1775. These changes point to a biological explanation, that the dominant smallpox viral strain circulating in England in the late eighteenth century became more infectious. An increase in infectiousness (probably accompanied by an increase in virulence) would have accelerated the spread of smallpox into rural populations, and also among London's children.

If correct, these findings have substantial implications for our understanding of mortality changes in the eighteenth century, and provide partial answers to the questions posed at the beginning of this article regarding the contribution of smallpox to mortality decline in both rural and urban populations in the late eighteenth century. With respect to London, the decrease in smallpox mortality evident in the London Bills in the late eighteenth century is unlikely to represent a genuine reduction in smallpox exposure or lethality. Rather it was probably the result of a rapid decline in the number of burials of adult migrants, with no improvement in smallpox mortality at younger ages. Since adult migrants comprised a large proportion of the London population, a decrease in their susceptibility, caused by higher rates of infection in childhood, and the increasing use of inoculation, would have reduced the smallpox burial totals, without any change in the rate of infant and child smallpox mortality, because susceptible adults were a distinct subpopulation, derived from immigrants to London.^86^ After 1800 a further rapid drop in smallpox burials coincided with the introduction of vaccination, and it was probably at this point, but not earlier, that London-born infants and children would have begun to escape the inevitability of smallpox infection. For young adults, this change occurred in the last quarter of the eighteenth century, and London became a substantially less dangerous place for adult migrants from this date.

Our evidence for continuing high levels of especially infant smallpox mortality before 1800 indicates that smallpox did not contribute to the rapid decline in infant mortality that appears to have occurred in London from at least the 1770s.^87^ While smallpox apparently accounted for only 4–7 per cent of infant mortality (figure 4b), it is likely that its effects were far greater, given both the potential for under-recording, and synergy between smallpox and other diseases. Smallpox caused debility in many survivors and may have contributed significantly to susceptibility to other diseases;^88^ its importance as a contributory cause of death is suggested by the large decline in infant mortality in the early 1800s that coincided with the introduction of vaccination. Assuming that infant mortality in St Martin's followed the trends evident in the London Bills, the explanation for the apparent paradox, of rising smallpox mortality and declining overall mortality, probably lies partly in the different trends of the various components of infant mortality. The decline of infant mortality nationally in the second half of the eighteenth century was driven almost totally by a decline in mortality in the first few months of life, ages when smallpox was not a major contributory cause. For London, where the decline in infant mortality was more dramatic, the only evidence for the relative contributions of endogenous and infectious factors derives from Landers's Quaker family reconstitutions. The Quaker data indicate a large decline in endogenous infant mortality, and a smaller decline in mortality of older infants and children (the latter in contrast to the stubborn persistence of rates at these ages in the Cambridge Group sample). A similar decline in the non-Quaker population of London seems unlikely, given our evidence for rising infant smallpox mortality, and the paucity of evidence for widespread inoculation in London. One possibility is that the London Quakers showed greater zeal for inoculation than the majority of Londoners. A number of prominent advocates of inoculation were Quakers (including Dimsdale, Fothergill, and Lettsom), and private inoculation may have been common among urban Quakers.^89^ Therefore it seems possible that infant mortality in London declined in the late eighteenth century *despite* an increase in smallpox mortality, and mainly as a consequence of improvements in neonatal mortality. It should be noted, however, that if smallpox incidence did indeed rise in infancy and early childhood, as suggested here, then the apparent absence of a *rise* in exogenous mortality at these ages would imply declines in other exogenous causes, sufficient to offset the impact of smallpox.

The simultaneous decline in smallpox risk of young adults and endogenous infant mortality raises the fascinating question of whether the two were related, through some effect on maternal health. Endogenous infant mortality rates in urban and rural areas converged over the late eighteenth century, despite persistent differences in environmental conditions, suggesting the common influence of some factor unrelated to living conditions.^90^ Recently, Woods has proposed that smallpox was a major contributor to both maternal and foetal mortality, and that a decline in smallpox in the late eighteenth century produced a parallel decline in both series (and by implication in early neonatal mortality as well).^91^ This is a fascinating hypothesis, and superficially fits our own data and the evidence from the London Bills of Mortality extremely well, since the downturn of ‘childbed’ and ‘stillborn’ rates in the Bills coincides very neatly with the abrupt decline in adult smallpox risk in St Martin's. However, it seems unlikely that smallpox was ever sufficiently common in pregnancy to contribute substantially to stillbirth or maternal mortality rates. Woods's model is predicated on both high levels of susceptibility among women of reproductive age, and high attack rates, in order to produce a significant effect of smallpox on either maternal or foetal mortality.^92^ However, since these were typical only where smallpox epidemics were rare, then such events would have been too infrequent to make a substantial impact on long-run foetal or maternal mortality rates. Even in London, with a relatively high proportion of susceptible adults, the proportion of women at risk, and the chances of infection specifically in later pregnancy, are unlikely to have been high enough to create the effect hypothesized by Woods; certainly the evidence from sex ratios of adult smallpox burials in St Martin's does not suggest that pregnant women were at special risk. Nevertheless, it remains plausible that a reduction in the incidence of adult smallpox contributed to improvements in maternal health and consequent improvements in foetal and neonatal health.

With respect to the impact of smallpox on death rates outside the metropolis, our analysis indicates, together with Razzell's evidence of a north–south divide, a geographically uneven pattern of smallpox epidemics and susceptibility in the period before 1775. After 1775 the disappearance of young adult smallpox victims suggests that the average age of smallpox infection probably fell everywhere, contributing to further declines in mortality of young adults and older children, but increasing the smallpox risk at the youngest ages. However, it is clear that inoculation was popular in many rural areas, and this may have been sufficient to counter any rise in smallpox infectiousness. Therefore a genuine reduction in smallpox risk may have occurred at *all* ages in rural populations that could not be detected in our London data. In this respect it is curious that the timing of declines in age-specific mortality rates from the Cambridge Group reconstitution sample shows little evidence of any impact of either inoculation or vaccination.^93^ This may reflect the geographical heterogeneity of both smallpox mortality and preventative measures. Nevertheless it appears that the impact of smallpox was greatest in urban populations, and that the greater relative improvement in urban death rates compared with rural owed much to the decline in smallpox, from *c.* 1770 for adults and older children, and from *c.* 1800 for infants. In rural areas smallpox was never such a major cause of death, and preventative measures were probably adopted over a more protracted period, with less striking results. The differential uptake of inoculation between urban and rural populations also raises the question of whether class differences in the adoption of inoculation could have contributed to the emergence of class differences in life expectancy which seems to have occurred in this period. The early decline of smallpox among the London Quakers is suggestive in this respect.

## Footnotes

2 Wrigley, ‘Simple model’; Schwarz, *London*, pp. 130–4; Landers, *Death*, pp. xix–xxi, 351–7; Galley, ‘Model’.

3 Hollingsworth, ‘Mortality’; Smith and Oeppen, ‘Place and status’; but see also Razzell and Spence, ‘Hazards’.

4 Wrigley, Davies, Oeppen, and Schofield, *English population*, pp. 12–16, 210–353.

5 City of Westminster Archives Centre (hereafter CWAC), London, Accession 419/123, 233–244, F2469, St Martin-in-the-Fields, sextons' day books.

6 Creighton, *History*, p. 544; Razzell, *Conquest*, pp. 172–8; but see Rutten, ‘*De vreselijkste*’, p. 417, for evidence that smallpox was unlikely to have killed more than 13% of sufferers in the eighteenth century.

7 Duncan, Scott, and Duncan, ‘Dynamics of smallpox epidemics’, pp. 409–10; Duncan, Duncan, and Scott, ‘Oscillatory dynamics’, pp. 449–50.

8 Landers, *Death*, pp. 153–9.

9 Razzell, *Conquest*, pp. 17–19, 79–94.

10 Ibid., pp. 94–6.

11 See section IV.

12 Landers, *Death*, pp. 152–6. Landers used all six London Quaker meetings for his analysis of age distribution, and calculated mortality rates from family reconstitution of two meetings.

13 Ibid., pp. 154–5.

14 Ibid., pp. 139–42.

15 Wrigley et al., *English population*, pp. 217–79.

16 IIbid., pp. 268–79; Smith and Oeppen, ‘Place and status’, pp. 64–8.

17 Landers, *Death*, pp. 355–6.

18 The last period, 1750–99, therefore includes both the peak period of smallpox in London, and its early decline.

19 Landers, *Death*, pp. 139–41; Vann and Eversley, *Friends*, pp. 197–201.

20 Vann and Eversley, *Friends*, pp. 221–2, doubt this was the case, but present no evidence either way.

21 For England, see Razzell, *Conquest*; Mercer, ‘Smallpox’.

22 McKeown, *Modern rise*, pp. 11–13, 107–8.

23 Decadal burial totals were: 6,490 (1752–9), 8,462 (1760–9) 10,011 (1770–9), 10,203 (1780–9), and 9,267 (1790–9). See Schwarz and Boulton, ‘Yet another inquiry’, and J. Boulton, ‘Traffic in corpses: interment, burial fees and vital registration in Georgian London’, unpub. paper in preparation, for details of the compilation and purposes of the sextons' books. Causes of death appear to have derived from the searches conducted for returns to the Bills of Mortality.

24 Or rather from 1725, when St George Hanover Square was excised to form a parish of its own. The population can be estimated roughly from the annual baptism counts over the period by assuming a constant birth rate, such as 30 births/1,000 population. The census population was 25,752 in 1801. St Martin's comprised around 5% of burials in the Bills of Mortality in the eighteenth century.

25 Schwarz, ‘Hanoverian London’, pp. 95, 97–8.

26 This was the conclusion of a number of contemporary commentators on the Bills, as well as most recent scholars, although there is some disagreement on the extent of under-recording of infant smallpox deaths. See Creighton, *History*, pp. 530, 534; Razzell, *Conquest*, pp. 133–4.

27 Dixon, *Smallpox*, p. 324; Razzell, *Conquest*, pp. 133–40.

28 See note to tab. 1, and n. 66 below.

29 Adults aged 10–39 accounted for 17.8% of burials in the years 1752–66, and 16.6% in 1775–99, indicating that this age group probably remained fairly stable as a proportion of the population, and that there was no abrupt change in the level of migration into St Martin's.

30 Razzell, *Conquest*, p. 96; Woodville, *History*, vol. II, p. 231.

31 Razzell, *Conquest*, p. 176.

32 Schwarz, ‘Social class’.

33 Landers, *Death*, pp. 115–19.

34 Ibid., pp. 288, 297–8.

35 Contrary to these indirect forms of evidence from the London bills, Landers's analysis of the London Quakers did not show any evidence of a shift in age structure of smallpox mortality after the 1760s. However, any such shift would have been difficult to detect because the small numbers of burials involved precluded further division of the data beyond the 50-year periods 1700–49 and 1750–99. There was some evidence of a reduction in adult burials in the second period among deaths explicitly labelled as smallpox, but this disappeared when the deaths were adjusted for burials without recorded cause—see note to tab. 1.

36 Creighton, *History*, p. 533.

37 Razzell, *Conquest*, pp. x–xvi. ‘Children’ were defined as under 21, because parish records often recorded whether a burial was of a child or man/woman, but gave no further details of age. However, variations in usage of ‘child’ in burial records may explain some of the patterns observed (see note to tab. 1).

38 Creighton, *History*, pp. 527, 536. See also Duncan et al., ‘Dynamics of smallpox epidemics’, pp. 410–12; Duncan, Scott, and Duncan, ‘Smallpox epidemics in cities in Britain’, pp. 260–7.

39 Razzell, *Conquest*, pp. xi–xiii; Gani and Leach, ‘Transmission’.

40 R. Davenport, unpub. analysis of the dataset ‘1765 Smallpox Census’ obtained from the Shakespeare Birthplace Trust (adults estimated at a minimum as lone householders, or heads of households in which all members were enumerated as vulnerable). In addition, the source lists the settlement status of inhabitants, and analysis indicated no difference in risk between adults with and without settlement in the town.

41 Razzell, *Conquest*, p. xvii. Susceptibles were calculated as those infected plus those inoculated, and excluding those who escaped either type of infection, who were not enumerated.

42 Dobson, *Contours*, pp. 477–81.

43 Other examples are documented in Razzell, *Conquest*, pp. xi–xiii, tab. 6.

44 Wareing, ‘Changes’; idem, ‘Migration’.

45 Razzell, *Conquest*, pp. xvi, 127; Brunton, ‘Smallpox’, p. 409; Sköld, *Two faces*, p. 105.

46 It is possible that dispersed populations may experience more prolonged disease outbreaks than more concentrated settlements of the same population size, because susceptible individuals are infected at a slower rate and therefore more remain available to sustain transmission, making it possible for the disease to circulate without fresh introduction and to infect successive cohorts of susceptibles. Denser settlements may experience more acute epidemics, but may also avoid epidemics even when the population of susceptibles is sufficient to sustain an epidemic, if re-introduction of infection is haphazard; Cliff, Haggett, and Smallman-Raynor, *Island epidemics*, pp. 97–115. Brunton, ‘Smallpox’, p. 409, made this point with respect to Highland Scotland. See Dobson, *Contours*, pp. 477–9, for evidence of such a process in south-east England, and Rutten, ‘*De vreselijkste*’, pp. 111, 418, for evidence of high adult vulnerability in smaller settlements in the Netherlands.

47 In Sweden adults aged 10+ comprised 5–6% of smallpox deaths in the period 1776–1805; Sköld, ‘Inoculation’, p. 261.

48 This is because in an endemic situation the number of smallpox burials should decline exponentially with age (assuming similar age-specific case-fatality rates), so the age structure of the population would need to be distorted to an improbable degree by migration to account for the excess of young adult burials.

49 Quoted in Creighton, *History*, p. 533, n. 1.

50 Landers, ‘Mortality’; Galloway, ‘Annual variations’.

51 Duncan et al., ‘Oscillatory dynamics’.

52 Note that this implies a very high level of endemicity in London, because the age structure of the population was much older than that of national populations, due to high rates of adult immigration.

53 Meier, ‘Smallpox’, pp. 102, 107.

54 Wareing, ‘Migration’, pp. 372–3; Sharpe, ‘Population’, p. 497.

55 Stone, *Family*, p. 77, cites a would-be servant from Bletchley who had himself inoculated in 1766 ‘ “as he had a design to go to London for a place” and he knew that this was an essential requirement for employment in a genteel household’. For other examples, see Morris, ‘Smallpox’, p. 1261; Buchan, *Domestic medicine*, p. 232. See also section IV.

56 Wareing, ‘Migration’, p. 374, found on the contrary that indentured servants bound in London for service in the colonies had migrated greater distances to London on average than apprentices in the first half of the eighteenth century, but information regarding origins of different occupational groups in London is very fragmentary.

57 Razzell, *Conquest*, pp. 77–122. Brunton, ‘Smallpox’, also documented a rise in inoculation in Scotland from around 1770, although she thought the level of inoculation too low to have had much impact.

58 Quoted in Razzell, *Conquest*, pp. 113–14.

59 Anon., *Considerations*, p. 7. On arrival from South Carolina in 1771, Henry Laurens delayed his entry to London in order to have his family inoculated; Flavell, *London*, p. 14. See Razzell, *Conquest*, pp. xv–xvi, 150–1, for other examples of deliberate avoidance of smallpox infection.

60 Razzell, *Conquest*, p. 96. The hospital would not inoculate anyone under the age of seven.

61 Ibid., pp. 95–6; Rusnock, *Vital accounts*, pp. 95–100.

62 There were also the armed forces, where inoculation seems to have been rather an ad hoc affair, sometimes given to soldiers and sailors, sometimes not. In 1756 and 1775 sections of the Army were inoculated; see Kopperman, ‘British army’, pp. 69–70; and, for the Navy, a rather uncertain reference in Lincoln, ‘Medical profession’, p. 220.

63 We owe this information to Dr Alysa Levene. General inoculations may also have been conducted within the hospital when a smallpox epidemic threatened; Creighton, *History*, pp. 514–15.

64 Anon., *Sketch*, p. 4.

65 The decrease in adult susceptibles could have slowed the spread of smallpox within London and reduced the frequency of smallpox epidemics and thus childhood smallpox mortality by either process, but in fact this does not seem to have occurred.

66 Notably, infant mortality in St Martin's did not exhibit secular decline until around 1800. However fig. 4a is misleading in this regard, because infant burials were probably undercounted in the earlier period, 1752–66, due to the export of some corpses for extra-parochial burial, a practice which was not recorded before 1767; Boulton, ‘Traffic in corpses’ (in preparation). This phenomenon does not affect our analysis of smallpox mortality, because the pattern of exported burials by age and cause was very similar to that of burials in the parish, when exported burials could be distinguished from 1767. Exported burials included a slightly lower proportion of infants, and a slightly higher proportion of males, biases that would have acted to reduce the magnitude of the changes in age and sex structure of smallpox victims that we have reported here. We have excluded burials imported from other parishes from this analysis, but these were also very similar throughout the period in their distribution by age and cause to those with local addresses in the sextons' books, suggesting by analogy that the changes in recording of exported burials did not affect the relationship between smallpox and other causes of death. Note that pauper burials, which were not subject to export or import, displayed the same ratios of smallpox to all-cause burials, and changes in these ratios, as non-pauper burials. Further tests for potential artifacts arising from this problem are detailed later in the text.

67 See above, n. 66.

68 See above, n. 66.

69 Breastfeeding does not provide specific immunity to viral diseases such as smallpox, although it may reduce the ability of viruses and other pathogens to gain entry via the digestive and respiratory tracts. See, for instance, Hoshower, ‘Immunologic aspects’. Of course, neonates of non-immune mothers were particularly vulnerable, and some neonatal smallpox deaths will belong in this category, although the proportion of non-immune mothers presumably declined steeply after the 1770s.

70 Cited in Creighton, *History*, p. 534.

71 Mercer, *Disease*, pp. 63–4; Razzell, *Conquest*, pp. 133–41.

72 The average age of smallpox victims halved between the third and fourth quarters of the eighteenth century (tab. 1). However, it is not possible to establish the change in average age at death for the *London-born* population directly, since these are not identified in the source.

73 Anderson and May, *Infectious diseases*, p. 129.

74 Nicoara, Zäch, Trachsel, Germann, and Matter, ‘Decay’.

75 It was not possible to compare changes in smallpox burials at single years of age in Stepney, because until 1756 those aged under two years were recorded only as being children, and could not be separated from other child burials at older ages. From 1757 to 1773 age was poorly recorded.

76 Sköld, ‘Inoculation’, p. 261.

77 Foege, Millar, and Henderson, ‘Smallpox’; Arita and Wickett, ‘Impact’, pp. 868–9. Estimates of ‘R_o_’ values (a measure of the number of susceptibles infected by a single smallpox case, at the beginning of an outbreak) for smallpox are low compared to most epidemic diseases (around four to five); Anderson and May, *Infectious diseases*, p. 70; Gani and Leach, ‘Transmission’. However, Gani and Leach also suggest that R_o_ values could have been as high as 10–12 in the crowded conditions of eighteenth-century London. The average age at infection suggested by the age pattern of mortality is lower than implied by a biannual epidemic cycle, and supports the ‘forced’ models of Duncan et al. (see, for example, Duncan, Duncan, and Scott, ‘Oscillatory dynamics’).

78 Anderson and May, *Infectious diseases*, p. 89.

79 Dixon, *Smallpox*, pp. 5–8, 297–300.

80 Razzell, *Conquest*, pp. xi–xiii. See ibid., p. xxii, for his suggestion that lethality may have risen dramatically in the 1760s.

81 Ibid., p. 176.

82 Ibid., p. 190.

83 See above, n. 75.

84 Perrenoud, ‘Contribution’, p. 184.

85 Ibid., p. 186.

86 This change in the composition of the susceptible population may explain the absence of any shift in the inter-epidemic interval in this period; Duncan et al., ‘Oscillatory dynamics’. The inter-epidemic interval is generally modelled as a function of the size of the population of susceptibles, and the transmission rate. In the present case, a rise in transmission rate would have coincided with a fall in the number of susceptibles, which may have been sufficient to maintain a biennial cycle.

87 Landers, *Death*, p. 192.

88 Razzell, *Conquest*, pp. 140–5; Mercer, *Disease*, p. 73. This was widely thought at the time. However, the standard medical work on smallpox plays this down; Dixon, *Smallpox*, p. 104.

89 Lobo, ‘John Haygarth’.

90 Wrigley et al., *English population*, pp. 223–5; Smith and Oeppen, ‘Place and status’, pp. 62–70.

91 Woods, *Death*, pp. 217–32.

92 Ibid., pp. 229–30.

93 Wrigley et al., *English population*, pp. 250–1, 290–1.
